# Handheld ECG Tracking of in-hOspital Atrial Fibrillation (HECTO-AF): A Randomized Controlled Trial

**DOI:** 10.3389/fcvm.2021.681890

**Published:** 2021-05-04

**Authors:** Marco Mancinetti, Sara Schukraft, Yannick Faucherre, Stéphane Cook, Diego Arroyo, Serban Puricel

**Affiliations:** ^1^Department of General Internal Medicine, University and Hospital Fribourg, Fribourg, Switzerland; ^2^Department of Cardiology, University and Hospital Fribourg, Fribourg, Switzerland

**Keywords:** atrial fibrillation, ECG handheld device, prevention, screening, stroke

## Abstract

**Background:** Atrial fibrillation (AF) is frequent and causes substantial morbidity through AF-related strokes. Given the increasing prevalence of AF, screening methods are of interest given the potential to initiate timely appropriate anticoagulation.

**Aims:** The HECTO-AF trial aims to determine the efficacy of AF screening with a single-lead electrocardiogram (ECG) handheld device in naïve in-hospital patients.

**Methods:** The HECTO-AF is a single-center, open label, randomized controlled trial. Patients admitted to the general internal medicine ward of the University and Hospital Fribourg without previous diagnosis of AF were invited to participate in a screening program with a 1:1 allocation to either the screening group with intermittent single-lead handheld ECG recordings vs. a control group undergoing detection of AF as per routine clinical practice. The primary outcome was the prevalence of newly diagnosed AF during the hospital stay. Enrolment was terminated for poor patient recruitment and apparent futility before a sufficient sample for powered efficacy comparisons was enrolled.

**Results:** A total of 804 patients were included of whom 381 were allocated to the intervention and 423 to the control group. Mean age was 65 ± 16 and 464 (58%) were male. Median CHA_2_DS_2_-VASc score was 3 (13% heart failure, 57% hypertension, 19% diabetes mellitus, 14% prior stroke/transient ischemic attack, and 29% arterial disease) and all CHA_2_DS_2_-VASc risk factors were equally distributed between groups. The incidence of newly detected AF was 1.4% over a median of 6 hospitalized days. Seven patients (1.8%) were diagnosed with AF in the intervention group vs. 3 (0.7%) in the control group (*p* = 0.20).

**Conclusion:** There was a trend toward a higher AF detection over a median of 6 hospitalized days in the intervention group, but a definitive conclusion cannot be drawn due to the early termination of the present study. Systematic screening for AF in the hospital setting is resource-consuming, and of uncertain clinical benefit. The interpretation of single-lead handheld ECG is challenging and may result in inaccurate AF diagnosis.

**Clinical Trial Registration:** ClinicalTrials.gov, identifier [NCT03197090].

## Introduction

Atrial fibrillation (AF) affects over 5 million people in Europe, with projected estimates up to 14 million by 2060 making it the most common arrhythmia (1). AF patients have a two-fold increase in mortality and a five-fold increase in risk of stroke compared with the general population (2). It is estimated that one third of AF patients will be hospitalized at least once a year due to worsening heart failure or cardioembolic events (3). Asymptomatic or “silent” AF is present in close to 33% of patients and conveys a risk of stroke identical to symptomatic patients (2). There is evidence that even short episodes of silent AF (of at least 6 min) have an increased thromboembolic risk (4).

The 2016 European Society of Cardiology (ESC) guidelines encourage opportunistic AF screening programs in at-risk populations by pulse palpation (5). To date there is insufficient evidence for a systematic screening strategy (6). However, a number of randomized controlled trials have investigated the effectiveness of routine screening for AF in outpatients using different devices. Among them, is the Zenicor single-lead electrocardiogram (ECG) recording device which appropriately detects AF with a sensitivity of 96%, and a specificity of 92% (7). Data on AF screening strategies and clinical benefit in hospitalized patients are lacking.

HECTO-AF is the first study to assess the effectiveness of systematic screening for silent-AF using a single-lead ECG handheld device vs. routine clinical practice in patients hospitalized in the general internal medicine ward.

## Methods

In this single center, open label, randomized controlled trial, we randomly assigned patients in a 1:1 ratio to a systematic screening strategy using the Zenicor (Medical Systems AB) single-lead handheld ECG recording device vs. a control group with standard clinical care. The study methods and design have been published previously (8). The study was conducted in accordance with the Declaration of Helsinki and was approved by the regional ethics committee (ClinicalTrials.gov, ID: NCT03197090, first registration on 23/06/2017). All patients provided written, informed consent for participation.

**Figure 1:**
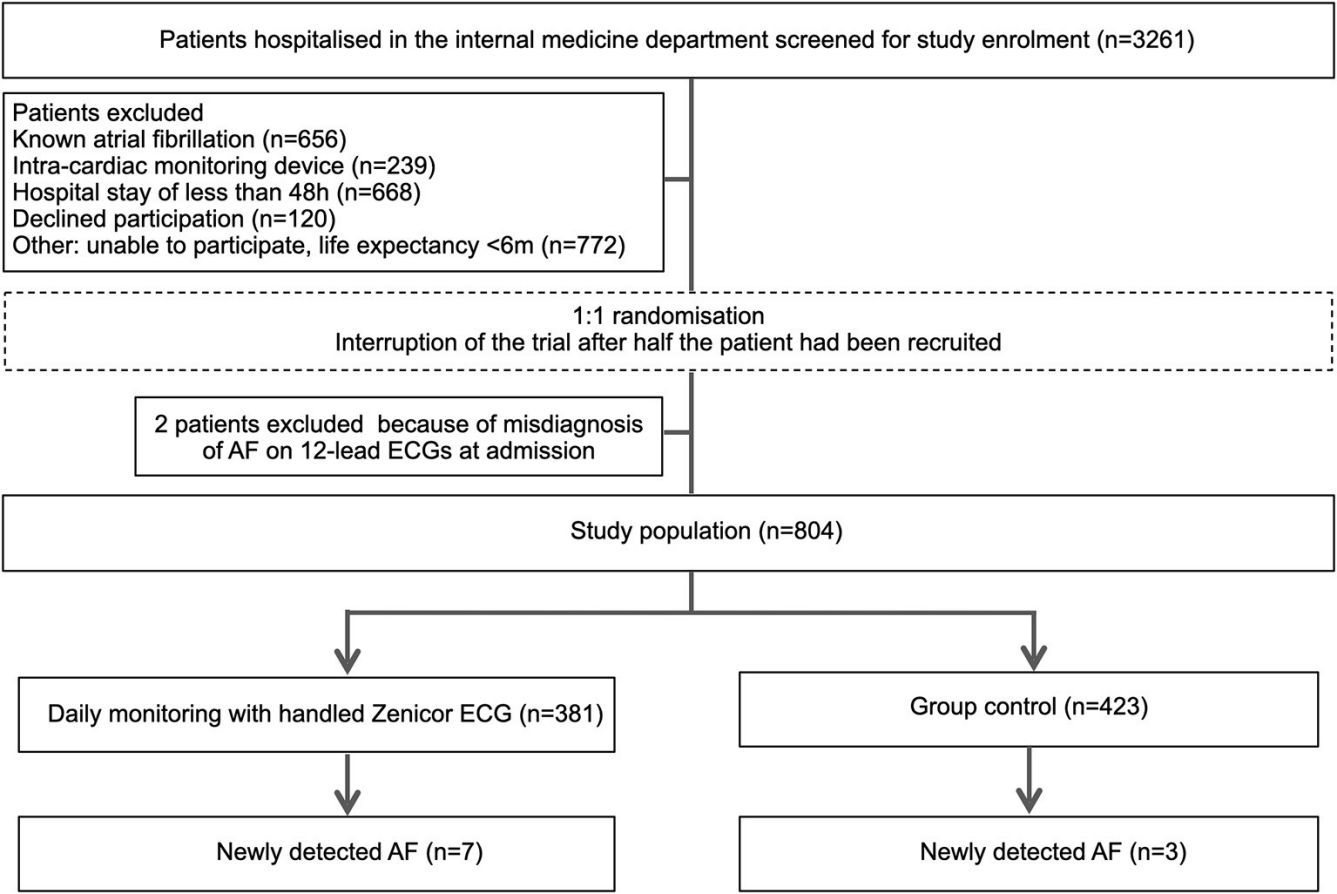
Study flowchart. Participants are 1:1 randomized to either handheld single-lead ECG (Zenicor) screening or control group.

All patients 18 years or older admitted to the general internal medicine ward were eligible. Patients with known or previously documented AF, patients with cardiac pacemakers or implantable cardioverter-defibrillators, length of stay <48 h, life expectancy <6 months, and those unable to provide written-informed consent, were excluded (Figure 1).

Randomization was performed as soon as written consent was provided, using a computer-generated allocation sequence (www.randomizer.org) and concealment until assignment. Research nurses generated the random allocation sequence, enrolled participants and assigned the patients to the study groups according to the previously generated randomization sequence. There was no blinding.

### ECG Recording

Patients included in the Zenicor group were instructed to use the handheld ECG recorder for intermittent ECG recordings during the hospitalization period. Recordings were planned twice daily under supervision of specially trained nurses. Patients had to apply their thumbs over the captors of the device during 30-s to yield a single-lead ECG recording which was stored, and subsequently transmitted to a central server for analysis. In the control group, 12-lead ECG were performed and interpreted by the treating physicians as per standard clinical care (i.e., in case of palpitations, chest pain, suspicion of arrhythmia during physical examination).

### ECG Analysis

**Figure 2:**
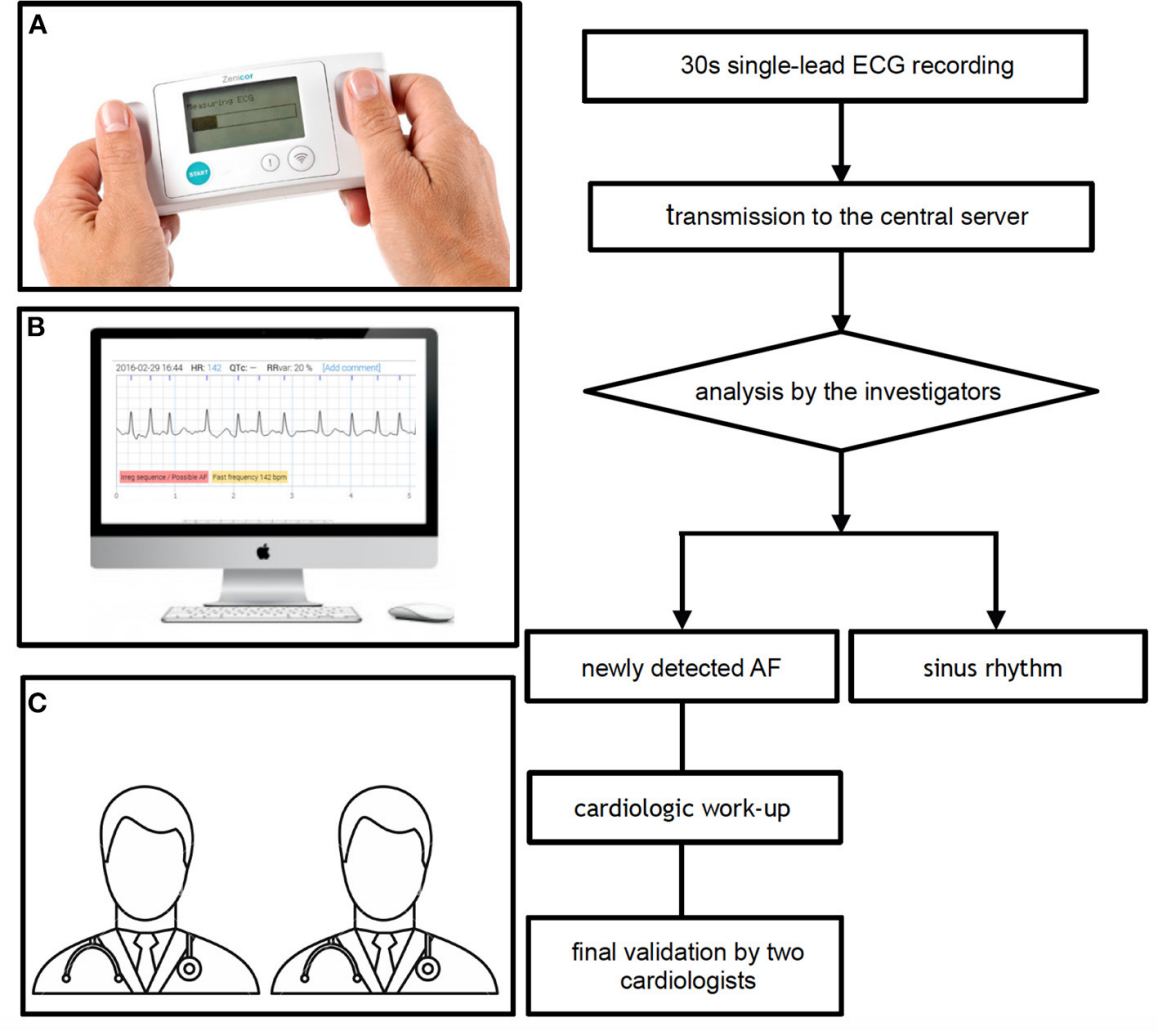
Single-lead ECG recording and interpretation with the Zenicor device. **(A)** Thirty second single-lead ECG handheld device recording, **(B)** analysis of the ECG on the central server, and **(C)** final validation by two cardiologists in case of newly detected AF. AF, atrial fibrillation; ECG, electrocardiogram.

All single-lead ECGs were stored in a web-based interface analysis system (Zenicor-ECG Doctor System) and independently reviewed on the same day by the investigators to assess the presence of AF (Figure 2). A 12-lead ECG was performed in all cases of suspected AF in the Zenicor group. Additional recordings, including 24–48 h Holter monitoring or 7-day R-Test were performed in case of uncertainty. Finally, two cardiologists reviewed every case of suspected new AF.

### Outcomes

The primary outcome was the incidence of new-onset AF defined as a 30-s recording of irregular rhythm without p waves (5). Each patient with newly diagnosed AF was treated according to the 2016 ESC guidelines on the management of atrial fibrillation (i.e., regarding anticoagulation, rhythm, and/or rate control therapy). Additional workup including 12-lead ECG, laboratory investigations, and further cardiological workup such as echocardiography, were organized as per guidelines.

### Statistics

We calculated that a total of 1,600 patients would yield a power of 80% to detect superiority with an estimated event rate of 3% in the Zenicor group and 1% in the control group, allowing a 9% loss to follow-up. The trial was interrupted at interim analysis after 50% of patients were enrolled (*n* = 804), due to poor patient recruitment and limited resources. The premature interruption of the trial was responsible for patient number differences between groups. The inclusion of 804 patients yields a power of 53% for the presumed event rate of 3 and 1%, respectively. Because of the potential for type I error due to incomplete patient enrollment, the reported analyses should be interpreted as exploratory.

Categorical variables are reported as counts and percentages; continuous variables are reported as means and SD. Normality was assessed by visual inspection of histograms and computation of Q-Q plots. Continuous variables were analyzed using the Student *t*-test or the Wilcoxon rank-sum test according to their distribution. Categorical variables were compared using chi-square or Fisher exact test as appropriate. All statistical analyses were performed using dedicated software (Stata 14, College Station, Texas) at a 2-tailed significance level of alpha = 0.05.

## Results

### Participation

From March 2018 and August 2019, 3,261 patients were screened for eligibility of which 806 (25%) underwent randomization. Among the main exclusion criteria, 656 patients were known to have AF, 239 patients had a cardiac pacemaker or implantable cardioverter-defibrillator, 668 had a length of stay <48 h and 120 patients declined participation. While were was no systematic analysis of consent refusals, frequent reasons for declining participation were anxiety about being labeled with an unexpected diagnosis and the risk of undergoing further investigations. Two patients were excluded from the analysis because of AF on 12-lead ECGs at admission. A total of 804 patients were included of whom 381 were assigned to intermittent single-lead handheld ECG recordings, and 423 to routine clinical practice. Patient flow-chart is depicted in Figure 1.

### Patient Characteristics

Baseline characteristics are summarized in Table 1. Mean age was 65 ± 16 years and 464 (58%) were male. Median CHA_2_DS_2_-VASc score was three of which: 13% heart failure, 57% hypertension, 19% diabetes mellitus, 14% prior stroke/transient ischemic attack, and 29% vascular disease (including myocardial infarction, peripheral artery disease, and complex aortic plaque). CHA_2_DS_2_-VASc risk factors were equally distributed between groups. All main diagnoses were well-balanced between the groups (Table 2). The most common primary diagnosis was malignancy (14%). Moreover, 34 patients (8%) were hospitalized due to an ischaemic stroke in the Zenicor group vs. 24 patients (6%) in the control group (*p* = 0.08).

**Table 1:** Baseline patient characteristics.

	**Zenicor (*n* = 381)**	**Control group (*n* = 423)**	***p*-value**
Male	216 (57)	248 (59)	0.62
Age, years	66.16 ± 14.79	64.57 ± 17.03	0.56
Hypertension	219 (57)	229 (54)	0.35
Diabetes mellitus	71 (19)	78 (18)	1.00
Dyslipidemia	145 (38)	134 (31)	0.06
Smoker or previous smoker	175 (46)	177 (42)	0.26
Positive family history for cardiovascular disease	66 (17)	81 (19)	0.52
Ischemic heart disease	72 (19)	69 (16)	0.35
Congestive heart failure	48 (13)	49 (12)	0.67
Previous stroke	55 (14)	52 (12)	0.40
Vascular disease (MI, peripheral artery disease, complex aortic plaque)	112 (29)	119 (28)	0.70
Age (>75 years)	215 (33)	135 (32)	0.82
**CHA**_**2**_**DS**_**2**_**-VASc risk score (categorical)**			
0–1	121 (32)	141 (33)	0.65
2–4	181 (48)	203 (48)	0.94
>4	79 (21)	79 (19)	0.48
CHA_2_DS_2_-VASc risk score (quantitative)	2.80 ± 1.93	2.69 ± 1.89	0.52
Medication predisposing to bleeding (aspirin, clopidogrel, and NSAID)	224 (59)	226 (53)	0.14
**Length of hospitalization, days**			
Mean ± SD	10 ± 7	10 ± 8	0.78
Median (IQR)	8 (6–12)	8 (6–11)	

*Values are mean ± SD or n (%)*.

**Table 2:** Main diagnosis during hospitalization.

	**Zenicor (*n* = 381)**	**Control (*n* = 423)**	***p*-value**
Heart failure	19 (5)	21 (5)	1.00
Ischaemic heart disease	22 (6)	16 (4)	0.19
Pneumonia	31 (8)	43 (10)	0.33
Chronic obstructive pulmonary disease	12 (3)	8 (2)	0.27
Pulmonary embolism	15 (4)	6 (1)	0.28
Other lung disease	16 (4)	14 (3)	0.58
Ischemic stroke or TIA	34 (8)	24 (6)	0.08
Gastrointestinal bleed	11 (3)	12 (3)	1.00
Malignancy	47 (12)	65 (15)	0.22
Minor trauma	21 (5)	22 (5)	0.88
Vascular disease	6 (2)	6 (1)	1.00
Infection/sepsis	39 (10)	60 (14)	0.11
Kidney failure	8 (2)	5 (1)	0.40
Miscellaneous	100 (26)	121(29)	0.48

*Values are n (%)*.

*TIA, transient ischemic attack*.

### Atrial Fibrillation Screening in the Zenicor Group

Systematic single-lead ECG screening was performed in 381 patients. Although single-lead ECG were scheduled twice daily, compliance with study protocol was incomplete. Median number of ECG recordings per individual was 6 (IQR: 3–10), over a median screening period of 6 days (IQR: 4–10). A total of 3,015 records were made using the Zenicor device. The time required for the screening strategy was 1 h per patient for identification of inclusion/exclusion criteria, explanation of device use and recording of one-lead ECGs. The time required for post-recording interpretation of the ECG was variable (between 1 and 5 min per ECG), requiring single-lead ECG review 7 days a week.

### Primary Outcome

The overall incidence of newly detected AF was 1.4% (*n* = 10) over the 17 months inclusion period. A total of 7 AF episodes were detected in the Zenicor group vs. 3 AF events in the control group (1.8 vs. 0.7%, *p* = 0.20).

### Management of Newly Detected AF

Oral anticoagulation was initiated in seven newly detected AF patients with a median CHA_2_DS_2_-VASc score of 3 (1–4), of which four in the Zenicor group, and three in the control group. Amongst the three non-anticoagulated patients in the Zenicor group [median HAS-BLED score of 2.5 (1.5–4)], OAC therapy was withheld because of risk of fall, risk of bleeding, and advanced malignancy. History and follow-up of patients with newly detected AF is summarized in Table 3.

**Table 3:** Clinical characteristics of patients with newly detected atrial fibrillation.

**Patient**	**AF detection group**	**Main diagnosis**	**Possible trigger factor**	**Time to detection (days)**	**OAC**	**Type of AF**	**CHA2DS2-VASc/ HAS-BLED**	**Relevant FUP**	**Time to FUP (months)**
82 years-old women	Control	Interstitial pneumonia	Unknown	7	Yes	Paroxysmal	4/2	Ischemic stroke after OAC interruption (reason for interruption unknown)	12
83 years-old women	Control	Lumbar trauma	Hyperthyroidism	7	Yes	Paroxysmal	4/2	No known AF recurrence	6
80 years-old women	Control	Kidney failure	Unknown	5	Yes	Paroxysmal	6/4	No known AF recurrence	7
50 years-old man	Zenicor	Skin infection	Infection	1	Yes (for planned electrical cardioversion, but not long term)^*^	Paroxysmal	0/0	No known AF recurrence	12
74 years-old man	Zenicor	Pneumonia	Unknown	2	Yes	Paroxysmal	1/2	No known AF recurrence	8
78 years-old man	Zenicor	Gastro-intestinal bleeding	Bleeding	0	Yes (after resolution of gastrointestinal bleeding)	Paroxysmal	3/3	Death from septic shock	5
74 years-old man	Zenicor	Malignancy	Mild hypokalaemia, severe hypomagnesemia	1	No, advanced malignancy	Paroxysmal	1/3	Death from malignancy	1
90 years-old man	Zenicor	Cholecystitis	Infection, mild hypokalaemia	9	No, high risk of fall	Paroxysmal	3/3	Pulmonary embolism requiring OAC	1
88 years-old women	Zenicor	TIA	n/a	1	Yes	Paroxysmal	7/5	AF recurrence	24
68 years-old man	Zenicor	Malignancy	Mild hypomagnesemia	0	No, bleeding risk (esophageal cancer)	Paroxysmal	2/2	No known AF recurrence	12

*OAC, oral anticoagulation; TIA, transient ischemic attack; FUP, follow-up*.

* *instead, patient had spontaneous cardioversion and anticoagulation was stopped at 1 month in the absence of AF recurrence after 24-h Holter*.

### Harms and Misdiagnosis of AF

A total of four patients in the Zenicor group were initially considered to have AF, but were subsequently reclassified as no AF by two independent cardiologists. None of the latter were started on OAC as two were already anticoagulated for other indications (mesenteric venous thrombosis, pulmonary embolism), and two refused anticoagulation. All patients were correctly reclassified in the final analysis.

## Discussion

The HECTO-AF trial was designed to determine whether a systematic screening strategy using daily recordings with a single-lead handheld ECG device increases the detection rate of AF compared to standard clinical practice in the hospital setting. The main findings of the HECTO-AF randomized trial are: (a) the overall incidence of newly detected AF was 1.4% over a median of 6 hospitalized days; (b) in the systematic screening group, a total of seven AF episodes were detected of which 4 (57%) were started on OAC; (c) the systematic screening for AF in the hospital setting is resource-consuming, and of uncertain clinical benefit.

### Rational for AF Screening

The European Society of Cardiology Guidelines recommend opportunistic screening of AF using pulse palpation based on a randomized controlled trial which found 1.64% incidence of new AF in patients >75 years-old (9). Recently a number of studies have assessed the effect of systematic screening on the detection of AF, with the idea that even brief (30 s or longer) episodes of AF detected during a limited period are clinically relevant. Four randomized controlled trials (RCTs) have compared screening programs to routine care. The Screening for Atrial Fibrillation in the Elderly (SAFE) trial including 14,802 patients demonstrated that active screening for AF (invitation for a 12-lead ECG) was more effective than routine care (pulse palpation and ECG in case of pulse irregularity). This study conducted in 50 primary care centers in England found a detection rate of new AF of 1.63% a year in the intervention group vs. 1.04% in the control group (difference 0.59%, 95% CI: 0.20–0.98) (10). Another RCT comparing opportunistic pulse palpation to systematic screening with 12-lead ECG in outpatient primary care, reported, in 3,001 patients, a greater AF detection rate in the intervention group (4.5 vs. 1.3%, OR, 3.7; 95% IC: 2.2–1.6) (11). In the REHEARSE-AF Study which randomized patients with CHA_2_DS_2_-VASc score ≥2 and ≥65 years old, to an AliveCor Kardia monitoring vs. routine care, in 1,001 patients, showed a statistically significant increase in detection of AF in the monitoring arm over a 12-month period (3.8 vs. 0.9%, HR, 3.9; 95% IC: 1.4–10.4; *P* = 0.007) (12). Finally, the STROKESTOP trial (Systematic ECG screening for Atrial Fibrillation Among) has reported a prevalence of AF of 3% in 7,625 outpatients from Sweden undergoing a 2-week intermittent recording using the Zenicor device (13).

More recently, there has been a paradigm shift in clinical trial design with the Apple Heart Study, in which photoplethysmography was used to detect irregularity in 419,297 participants wearing the Apple Watch (14). Of these, 2,161 participants (0.52%) received a notification of pulse irregularity, and were assessed for the necessity to wear a 7-day ECG patch. Of those notified and wearing the ECG-patch, 153 patients were diagnosed with AF (0.036% of the total population). It must be stressed that Apple Watch-like devices attract younger populations with uncertainty about the clinical value of detecting AF in low-risk individuals.

The place of AF screening is widely debated, and despite meta-analyses pointing to an apparent benefit in patients > 40 years-old (15), a recent US Preventive Services Task Force (USPSTF) has concluded that the evidence on the benefits for AF screening with ECG is insufficient (16).

### Screening for Atrial Fibrillation in the Hospital Setting

HECTO-AF is the first randomized study to assess a systematic screening strategy using a handheld device in the hospital setting. The incidence of newly diagnosed AF episodes (1.8%, *n* = 7) in the Zenicor group was lower than expected in hospitalized patients compared to outpatients. Factors that could explain a lower detection rate in the internal medicine ward include a younger population, short hospitalization stay (median 6 days) resulting in shorter screening periods as compared with the 2-weeks in the STROKESTOP trial. All patients were considered to have paroxysmal AF, none required neither rhythm nor rate control.

### Recording and Interpretation of ECGs

Single-lead ECG recordings were performed at rest under direct supervision of nurses to ensure optimal quality. Nonetheless, single-lead ECG quality was variable, and in some situations uninterpretable. Poor quality has been described due to the electric disturbance caused by movements, or high thumb pressures during recordings (7). Overall, and after review by expert cardiologists, initial AF misdiagnosis was considered in four patients. 12-lead ECGs were performed in all cases of suspected AF in the Zenicor group. The most common reason for discrepancy between single-lead ECG and 12-lead ECG was the presence of atrial or ventricular premature beats.

As mentioned, a previous study in patients with known AF, calculated ECG Zenicor sensitivity at 96% and specificity 92% which points toward AF overdiagnosis (7). It must be stressed however, that this was done in a context of 10-s recordings and not 30 s. Our trial results also indicated potential AF overdiagnosis. Although our aim was not to test for sensitivity, nor specificity we hypothesize that they may have differed from previous reports.

### Potential Harms of AF Misdiagnosis

A recent meta-analysis suggests that there is a lack of data regarding potential harms of AF screening vs. no screening (15). Indeed, AF misdiagnosis can lead to the initiation of unnecessary treatment with potential complications, and unwarranted tests. We strived to reduce this harm by requiring confirmation of every suspected AF single-lead recording by two senior cardiologists. Additional 24-h Holter monitoring were necessary in four patients from the Zenicor group (57%) with suspected AF. None of them detected a recurrence of AF.

### Treatment of AF and Initiation of OAC Treatment in the Acute Setting

There is a lack of evidence on the benefit of anticoagulation initiation in newly diagnosed AF in the acute setting. Prior studies have shown that AF in sepsis is associated with higher in-hospital and 5-year stroke risk when compared with patients with no AF (17). Gundlund et al. (18) found a greater recurrence of AF among patients with infection-related AF and twice the risk of thromboembolic events compared to infections without AF at 1 year's follow-up. Conversely, a retrospective study (19) did not demonstrate a lower risk of ischemic stroke following anticoagulation in patients with new-onset AF associated with sepsis, acute pulmonary disease and myocardial infarction. Meanwhile, anticoagulant use was associated with a higher risk of bleeding in patients with acute pulmonary disease (6.8 vs. 11.8%, *p* <0.05). In our study, anticoagulation was not started in 3 (43%) patients in the intervention group because of bleeding risk. Overall, the uncertainty of initiating long-term OAC in the acute setting, limits the indication for systematic AF screening in the hospital setting. Finally, AF was paroxysmal in all patients, and none required rhythm nor rate control therapy on the long term.

## Limitations

The most important study limitation was the lack of statistical power caused by prematurely discontinuing patient enrolment. Bias may have occurred due to fluctuations of AF detection during the study. The time to inclusion was not standardized for each patient and may have led to underdetection of AF in patients with very short hospital stays. There was a potential selection bias as only patients capable of performing a proper single-lead handheld recording were eligible. Therefore, the most vulnerable and fragile patients who may have been at even higher risk of AF were excluded. This may have underestimated the overall AF rate, but the benefit of introducing OAC in this population is debatable. In some cases, the presence of artifacts has limited the interpretation of ECG. The sensitivity and specificity as well as positive and negative predictive values in the in-hospital setting were not assessed as no systematic simultaneous 12-lead ECG were performed with each one-lead ECG. Finally, this was a monocentric study with results that may not be applicable to a more general population.

## Conclusion

There was a trend toward a higher AF detection over a median of 6 hospitalized days in the intervention group, but a definitive conclusion cannot be drawn because of the insufficient statistical power of the present study. A systematic screening program with daily single-lead handheld ECG recordings is resource-consuming. The interpretation of single-lead handheld ECG is challenging and may result in inaccurate AF diagnosis. The long-term benefit of oral anticoagulation in patients with accurate detection of AF during acute illness is uncertain.

## Data Availability Statement

The raw data supporting the conclusions of this article will be made available by the authors, without undue reservation.

## Ethics Statement

The studies involving human participants were reviewed and approved by Cantonal Ethics Committee of Vaud (CER-VD). The patients/participants provided their written informed consent to participate in this study.

## Author Contributions

MM and SC conceived the original idea and planned the study. YF contributed to the implementation of the research and data management. SS contributed to the study design, analyzed the data, and wrote the manuscript with support from DA, SP, and MM. DA and SP contributed to data analysis. SC supervised the project. All authors contributed to the article and approved the submitted version.

## Conflict of Interest

The authors declare that the research was conducted in the absence of any commercial or financial relationships that could be construed as a potential conflict of interest.

## Abbreviations

AF: atrial fibrillation
ECG: electrocardiogram
FUP: follow-up
HECTO-AF: Handheld ECG Tracking of in-hOspital Atrial Fibrillation
IQR: interquartile range
NSAID: nonsteroidal anti-inflammatory drug
OAC: oral anticoagulation
SD: standard deviation
TIA: transient ischemic attack.
