# Supplementary material for: Handheld ECG Tracking of in-hOspital Atrial Fibrillation (HECTO-AF): A Randomized Controlled Trial
Source: Front Cardiovasc Med. 2021 May 4;8:681890. doi: 10.3389/fcvm.2021.681890 (PMC8129539; doi:10.3389/fcvm.2021.681890)
Supplement: Supplementary file 1 [file Data_Sheet_1.PDF]

DETECTION OF ATRIAL FIBRILLATION IN THE HOSPITAL SETTING BY USE OF A HANDHELD ECG RECORDING DEVICE

The HECTO-AF Trial

Clinical Study Protocol

This is a single center, open label, randomized control trial to assess the efficacy of a one-lead ECG handheld device (Zenicor) for the detection of previously unknown atrial fibrillation (AF) in hospitalized patients.

Study Type:	Clinical trial with Investigational Medicinal Product (IMP)
Study Categorisation:	Type A
Study Registration:	ClinicalTrial.gov
Study Identifier:	NCT03197090
Sponsor:	Fonds Scientifique Cardiovasculaire Fribourg
Principal Investigator:	Marco Mancinetti
Investigational Product:	Zenicor
Protocol Version and Date:	Version number 4.0 (13.08.2019)
Study number:	ClinicalTrials.gov ID: NCT03197090
Study Title:	Detection of Atrial Fibrillation in the hospital setting by use of a handheld ECG recording device

CONFIDENTIAL
The information contained in this document is confidential and the property of the Hospital and University of Fribourg. The information may not - in full or in part - be transmitted, reproduced, published, or disclosed to others than the applicable Competent Ethics Committee(s) and Regulatory Authority(ies) without prior written authorisation from the sponsor except to the extent necessary to obtain informed consent from those who will participate in the study.

Principal Investigator:
I have read and understood this trial protocol and agree to conduct the trial as set out in this study protocol, the current version of the World Medical Association Declaration of Helsinki, ICH-GCP guidelines or ISO 14155 norm and the local legally applicable requirements.

Principal Investigator:

I have read and understood this trial protocol and agree to conduct the trial as set out in this study protocol, the current version of the World Medical Association Declaration of Helsinki, ICH-GCP guidelines or ISO 14155 norm and the local legally applicable requirements.

Principal Investigator Fribourg le 20.11.2019 Marco Mancinetti

The Sponsor-Investigator have approved the protocol version 4.0 (13.08.2019) and confirm hereby to conduct the study according to the protocol, current version of the World Medical Association Declaration of Helsinki, ICH-GCP guidelines or ISO 14155 norm if applicable and the local legally applicable requirements.

Sponsor-Investigator Be, 26/11/19 Stéphane Cook

Table of Contents

STUDY SYNOPSIS 6

STUDY SUMMARY IN LOCAL LANGUAGE (FRENCH) 10

ABBREVIATIONS 13

STUDY SCHEDULE 14

1. STUDY ADMINISTRATIVE STRUCTURE 15

1.1 Sponsor, Sponsor-Investigator 15

1.2 Principal Investigator(s) 15

1.3 Statistician ("Biostatistician") 15

2. ETHICAL AND REGULATORY ASPECTS 16

2.1 Study registration 16

2.2 Categorisation of study 16

2.3 Competent Ethics Committee (CEC) 16

2.4 Ethical Conduct of the Study 16

2.5 Declaration of interest 16

2.6 Patient Information and Informed Consent 17

2.7 Participant privacy and confidentiality 17

2.8 Early termination of the study 17

2.9 Protocol amendments 17

3. BACKGROUND AND RATIONALE 18

3.1 Background and Rationale 18

3.2 Investigational Product and Indication 19

3.3 Clinical Evidence to Date 20

3.4 Medical Device: Rationale for the intended purpose in study (pre-market MD) 20

3.5 Explanation for choice of comparator (or placebo) 20

3.6 Risks / Benefits 20

3.7 Justification of choice of study population 21

4. STUDY OBJECTIVES 21

4.1 Overall Objective 21

4.2 Primary Objective 21

4.3 Secondary Objectives 21

5. STUDY OUTCOMES 21

5.1 Primary Outcome 21

5.2 Secondary Outcomes 21

6. **STUDY DESIGN** 22
6.1 General study design and justification of design 22
6.2 Methods of minimising bias 22
6.2.1 Randomisation 22
6.2.2 Blinding procedures 22

7. **STUDY POPULATION** 23
7.1 Eligibility criteria 23
7.2 Recruitment and screening 23
7.3 Assignment to study groups 23
7.4 Criteria for withdrawal / discontinuation of participants 23

8. **STUDY INTERVENTION** 24
8.1 Identity of Investigational Products 24
8.1.1 Experimental Intervention 24
8.1.2 Control Intervention 24
8.1.3 Packaging, Labelling and Supply (re-supply) 24
8.2 Medical Device Accountability 24
8.3 Return or Destruction of Medical Device 24

9. **STUDY ASSESSMENTS** 25
9.1 Study flow chart(s) / table of study procedures and assessments 25
9.2 Assessments of outcomes 26
9.2.1 Assessment of primary outcome 26
9.2.2 Assessment of secondary outcomes 26
9.3 Procedures at each visit 26
9.3.1 Baseline visit 26
9.3.2 Daily visit 27
9.3.3 Cardiological visit 27
9.3.4 Follow up 27
10. **SAFETY** 27
10.1 Definition and Assessment of safety related events 27
10.2 Reporting of Safety related events 27

11. **STATISTICAL METHODS** 28
11.1 Hypothesis 28
11.2 Determination of Sample Size 28
11.3 Planned Analyses 28

11.3.1 Primary Analysis 28
11.3.2 Secondary Analyses 29
11.3.3 Interim analyses 29
11.3.4 Safety analysis 29
11.3.5 Deviation(s) from the original statistical plan 29
11.4 Handling of missing data and drop-outs 29

12. QUALITY ASSURANCE AND CONTROL 30
12.1 Data handling and record keeping / archiving 30
12.1.1 Case Report Forms 30
12.1.2 Specification of source documents 30
12.1.3 Record keeping / archiving 30
12.2 Data management 30
12.2.1 Data Management System 30
12.2.3 Analysis and archiving 31
12.2.4 Electronic and central data validation 31
12.3 Monitoring plan 31
12.4 Confidentiality, Data Protection 31
12.3.1 Data protection and Storage at Zenicor (SWEDEN) 32
12.3.2 Security of data transmission between Fribourg and Sweden 32

13. FUNDING AND SUPPORT 33
13.1 Budget 33
13.2 Funding 33

14. REFERENCES 34

15. APPENDICES 35

STUDY SYNOPSIS

**Sponsor-Investigator**	Investigator Initiated Research
**Study Title:**	Detection of Atrial Fibrillation in the hospital setting by use of a handheld ECG recording device
**Short Title / Study ID:**	HECTO-AF<br>NCT03197090
**Protocol Version and Date:**	Version number 4 (13.08.2019)
**Trial registration:**	ClinicalTrials.gov
**Study category and Rationale**	Clinical trials of medical devices of Category 2 A (with CE marking)
**Phase of development:**	Device Verification Study (Phase IV)
**Background and Rationale:**	Atrial Fibrillation (AF) is the most commonly encountered sustained arrhythmia in clinical practice. Due to shifting demographics and increasing prevalences of cardiovascular disorders predisposing to its occurrence, the prevalence of AF is constantly rising. Current evidence suggests that systematic and opportunistic screening for atrial fibrillation increases the rate of detection of new cases compared to conventional practice. Given that roughly 20% of ischemic strokes are attributable to AF, timely detection of its occurrence may lead to improved stroke prevention and thus to a decreased morbidity and mortality engendered by AF.
**Objective(s):**	The aim of this study is to assess the efficacy of a one-lead ECG handheld device (Zenicor) for the detection of previously unknown atrial fibrillation in hospitalized patients.
**Primary Outcome:**	Proportion of newly detected atrial fibrillation in percent.
**Secondary Outcomes :**	- Death<br>- Cardiovascular death<br>- Stroke, transient ischemic attack or systemic embolism<br>- Myocardial infarction<br>- Bleeding
**Study design:**	This is a single center, open label, randomized control trial with an allocation ratio of 1:1 conducted in University and Hospital Fribourg.
**Inclusion / Exclusion criteria:**	All patients admitted to the internal medicine department of the University and Hospital Fribourg throughout the study period will be screened for study enrollment. Patients with known or previously documented atrial fibrillation,

	patients with a cardiac pacemaker, and implantable cardioverter-defibrillator or intra-cardiac monitoring device will be excluded from the present trial. Patients unable or unwilling to provide written informed consent and those unable or unwilling to participate in the study procedures or follow-up will likewise be excluded. Patients with an estimated life expectancy of <6 months will be excluded.
**Measurements and procedures:**	Patients allocated to the treatment group will undergo twice daily monitoring with the handheld Zenicor ECG. Additional recordings will be obtained whenever patients notice palpitations. Recordings will be obtained in the presence of specially trained nurses. The recordings will then be validated through a web-based interface offered by Zenicor.<br>The one-lead ECGs will be reviewed by the investigating physicians through the Web-based analysis service (Zenicor-ECG Doctor System) assessing for the presence of atrial fibrillation.<br><br>Patients in whom AF is newly diagnosed during the trial will undergo a prespecified work-up consisting of thransthoracic echocardiography, blood analysis and risk stratification. For patients with non-valvular AF in whom oral anticoagulation is indicated according to the 2016 ESC-Guidelines, NOACs will be the treatment of choice.<br>All enrolled patients will be followed clinically at 2, 5 and 10 years to assess the occurrence of death, cardiovascular death, stroke or transient ischemic attacks, systemic embolism, myocardial infarction and bleeding.
**Study Product / Intervention:**	The study product in this trial is the Zenicor 1-lead handheld ECG recording device (www.zenicor.com). The device has been shown to have higher sensitivity for detection of AF than conventional 24-hour Holter (SN 96%, SP 92%).
**Control Intervention:**	Patients allocated to the control group will not undergo systematic monitoring of AF. In those patients, recording of 12 lead ECG or any other monitoring modalities will be employed according to routine clinical practice.
**Number of Participants with Rationale:**	Based on previous research, we assume to detect AF in 1% of patients in the control group and in 3% of patients in the intervention group. The inclusion of n=745 patients per group would yield a power of 80% to detect that difference. In order to account for ineffective measurements, post-inclusion drop-outs or other unforeseen drop-outs, we seek to include n=800 patients per group.
**Study Duration:**	Based on the current statistics pertaining to admissions and length of stay at our institution we estimate a total duration of 15 months for patient enrollment and the assessment of the primary end point. Clinical follow-up

	will then be performed up to 10 years after that date.
**Study Schedule:**	Month Year of First-Participant-In (planned): 01.03.2018<br>Month Year of Last-Participant-Out (planned): 31.03.2028
**Investigator(s):**	Name: Dr. Mancinetti M<br>Address: Chemin des Pensionnats 2, 1708 Fribourg<br>Telephone number: 026 426 71 11<br><br>Name: Dr Puricel S<br>Address: Chemin des Pensionnats 2, 1708 Fribourg<br>Telephone number: 026 426 71 11<br><br>Name: Dr Schukraft S<br>Address: Chemin des Pensionnats 2, 1708 Fribourg<br>Telephone number: 026 426 71 11<br><br>Name: Prof. Hayoz D<br>Address: Chemin des Pensionnats 2, 1708 Fribourg<br>Telephone number: 026 426 71 11<br><br>Name: Prof Cook S<br>Address: Chemin des Pensionnats 2, 1708 Fribourg<br>Telephone number: 026 426 71 11
**Study Centre:**	This is a single center study conducted at University and Hospital Fribourg.
**Statistical Considerations:**	Categorical variables will be reported as counts and percentages; continuous variables will be reported as mean and SD or as median with 25% to 75% interquartile range according to their distribution. Normality will be assessed by visual inspection of histograms, the computation of Q-Q plots and the shapiro-wilk test. Categorical variables will be compared using chi-square or Fisher's exact test as appropriate. Continuous variables will be analyzed using the Student's t-test or the Wilcoxon rank-sum test according to their distribution. Survival free from the occurrence of clinical end points will be compared using the log-rank test and plotted as Kaplan-Meier survival functions. All statistical analyses will be performed using dedicated software (Stata version 13, StataCorp LP, College Station, Texas) at a 2-tailed significance level of alpha = 0.05.
**GCP Statement:**	This study will be conducted in compliance with the protocol, the current version of the Declaration of Helsinki, the ICH-GCP or ISO EN 14155 (as far as applicable) as well as all national legal and regulatory requirements.

RESUME DE L'ETUDE

**Titre de l'étude**	Détection intra-hospitalière de la fibrillation atriale par un ECG portatif
**Titre bref**	Detect-AF
**Version du protocole et date**	Version numéro 4.0 (13.08.2019)
**Enregistrement de l'étude**	ClinicalTrial.gov
**Catégorie de l'étude**	Etude de catégorie A (avec marquage CE)
**Phase de développement**	Essai clinique de phase 4
**Contexte**	La fibrillation auriculaire (FA) est l'arythmie la plus fréquemment rencontrée dans la pratique clinique. Sa prévalence augmente constamment en raison de la transformation démographique et d'une augmentation de la prévalence des maladies cardiovasculaires. Les études suggèrent que le dépistage systématique de la FA augmente son taux de détection par rapport aux pratiques diagnostiques usuelles
**Objectif**	L'objectif de cette étude est d'évaluer l'efficacité de l'ECG portatif Zenicor pour la détection de la fibrillation auriculaire asymptomatique chez les patients hospitalisés.
**Critère de jugement premier**	Proportion de fibrillation atriale nouvellement détectée en pourcent
**Design de l'étude**	Detect-AF est un essai randomisé contrôlé avec un ratio d'allocation de 1: 1 mené dans l'université et l'hôpital de Fribourg.
**Critères d'inclusion/ exclusion**	Tous les patients admis au département de médecine de l'Université et de l'hôpital de Fribourg au cours de l'étude seront sélectionnés pour l'inscription à l'étude. Les patients avec une FA connue, porteurs d'un stimulateur ou d'un dispositif de surveillance cardiaque seront exclus de l'essai clinique. Les patients incapables d'accorder leur consentement écrit seront également exclus. Les patients dont l'espérance de vie est estimée à moins de 6 mois seront exclus.
**Mesures et procédures**	Les patients affectés au groupe de traitement effectueront un enregistrement du rythme cardiaque par l'ECG portatif Zenicor deux fois par jour (ou plus). Des enregistrements supplémentaires seront obtenus chaque fois que le patient ressent des palpitations.<br>Les enregistrements seront effectués par des infirmières spécialement

	formées et seront ensuite transférés sur une interface Web avant d'être analysés par les investigateurs de l'étude.
**Produit d'étude / Intervention**	Le produit d'étude est l'appareil d'enregistrement portable d'ECG<br>Zenicor à une dérivation (www.zenicor.com). Il a été montré que l'appareil a une sensibilité plus élevée qu'un holter de 24 heures pour la détection de la fibrillation atriale (SN 96%, SP 92%, rapport de vraisemblance 0,043 à 12,00).
**Contrôle**	Les patients affectés au groupe témoin ne subiront pas une surveillance systématique de la FA. Chez ces patients, l'enregistrement d'un ECG standard sera utilisé selon la pratique clinique habituelle.
**Nombre de participants**	Sur la base des précédentes études, la détection de la fibrillation atriale chez les patients du groupe témoin est estimé à 1% et à 3% chez les patients du groupe interventionnel. L'inclusion de n = 745 patients par groupe donnerait une puissance de 80% pour détecter cette différence. Afin de tenir compte de la présence de possibles mesures non valides, de patients perdus de vues, nous cherchons à inclure n = 800 patients par groupe.
**Durée de l'étude**	La durée d'enregistrement des éléctrocardiogrammes sera de 15 mois et la durée totale de l'étude est estimée à 10 ans pour assurer le suivit des participants
**Calendrier**	Début de l'étude prévu: 01.03.2018<br>Fin de l'étude prévue: 31.03.2028
**Investigateurs**	Dr. Marco Mancinetti (Principal Investigator)<br>Departement de Medecne Interne<br>Chemin des Pensionnats 2, 1708 Fribourg<br>Téléphone: + 41 26 426 71 11<br>E-mail: marco.mancinetti@h-fr.ch<br><br>Dr. Serban Puricel<br>Departement de Cardiologie<br>Chemin des Pensionnats 2, 1708 Fribourg<br>Téléphone: + 41 26 426 71 11<br>E-mail: serban.puricel @h-fr.ch

Dr. Sara Schukraft

Departement de Cardiologie

Chemin des Pensionnats 2, 1708 Fribourg

Phone: + 41 26 426 71 11

E-mail: sara.schukraft@h-fr.ch

Prof. Daniel Hayoz

Departement de Medecine interne

Chemin des Pensionnats 2, 1708 Fribourg

Téléphone: + 41 26 426 71 11

E-mail: daniel.hayoz@h-fr.ch

Prof. Setphane Cook

Departement de Cardiologie

Chemin des Pensionnats 2, 1708 Fribourg

Téléphone: + 41 26 426 71 11

E-mail: stephane.cook@unifr.ch

**Centres d'études**	Etude monocentrique réalisée à l'Université et à l'hôpital de Fribourg
**Considérations statistiques**	La distribution normale des valeurs sera testée et les valeurs seront comparées entre elles en utilisant le t-test ou chi-carré le cas échéant. Les valeurs seront exprimées en pourcentages et en moyenne ± DS. Les tests seront bilatéraux et une valeur $P < 0,05$ sera considéré comme significative
**Déclaration GCP**	Cette étude sera menée conformément au protocole, à la version actuelle de la Déclaration d'Helsinki, à l'ICH-GCP ou à la norme ISO EN 14155 ainsi qu'à toutes les exigences juridiques et réglementaires nationales.

Abbreviations

AF	Atrial fibrillation
CA	Competent Authorities
CEC	Competent Ethics Committee
ECG	Electrocardiogramme
GCP	Good Clinical Practice
IMP	Investigational Medicinal Product
MD	Medical Device
OClin	Ordonnance sur les essais cliniques dans le cadre de la recherche sur l'être humain
PI	Principal Investigator
SAR	Serious Adverse Reaction

STUDY SCHEDULE

	Patient information and inclusion[1]	*Intervention Period*	*Investigations*	*Follow-up*
Visit	*Once or twice*	*twice daily*	*In patients with newly diagnosed AF*	*2 years,<br>5 years,<br>10 years*
Time	*20 minutes*	*30 seconds twice daily (or more)*	*30 minutes*	
Patient information	*x*			
Informed Consent	*x*			
Baseline characteristics: age, gender, CHAD-VASC score etc.	*x*			
Randomisation	*x*			
ECG recordings		*x*		
Primary outcome		*x*		
Laboratory tests: TSH ,lipid profile etc.			*x*	
ECG, Echo			*x*	
Secondary outcome (clinical follow-up)				*x*

[1] A period of 24 hours of reflection will be granted to the patient for the constent process

1. STUDY ADMINISTRATIVE STRUCTURE

1.1 Sponsor

Fonds Scientifique Cardiovasculaire Fribourg

Board members: Stéphane Cook, Daniel Hayoz, Bruno de Weck

Chemin des Pensionnats 2, 1708 Fribourg

Phone: +41 26 426 71 11

1.2 Principal Investigator

Dr Marco Mancinetti

Chemin des Pensionnats 2, 1708 Fribourg /

Phone: +41 26 426 71 11

1.3 Co-investigators

Dr Serban Puricel

Chemin des Pensionnats 2, 1708 Fribourg

Phone; +41 26 426 71 11

Dr Sara Schukraft

Chemin des Pensionnats 2, 1708 Fribourg

Phone: +41 26 426 71 11

Prof. Daniel Hayoz

Chemin des Pensionnats 2, 1708 Fribourg

Phone: +41 26 426 71 11

Prof Stéphane Cook

Chemin des Pensionnats 2, 1708 Fribourg

Phone: +41 26 426 71 11

1.4 Statistician ("Biostatistician")

The biostatistician will be responsible for the protocol development, and data analysis.

Dr Serban Puricel

Chemin des Pensionnats 2, 1708 Fribourg

Phone: + 4126426 71 11

2. ETHICAL AND REGULATORY ASPECTS

The Detect-AF study will be conducted with regards to the ethical foundations of research including performance of 4 core principles: autonomy, beneficence, nonmaleficene and justice.
Informed consent allows for the confirmation of autonomy among all research participants. A document relaying all pertinent and relevant research information, such as risks and benefits will be submitted to the subjects. Subsequently, the participant will be given the opportunity to withdraw from the trial at any time and for any (or no) reason.
Beneficence is respected given that the trial aims at an increased detection of AF with subsequent treatment according to good clinical practice. Thereby, we seek to decrease morbidity and mortality arising from undetected AF and from ischemic stroke and other systemic embolsims in undetected AF. Given the non-invasive nature of the ECG recordings we belive that the present trial is in accord with the principal of nonmaleficene. Finally, all subjects will be treated equally. There will be no exclusion of certain categories of potential participants such as gender, race, socioeconomic status or any other characteristics.

2.1 Study registration

ClinicalTrials.gov ID: NCT03197090

2.2 Categorisation of study

Zenicor is a Category A medical device because it bears a conformity marking (CE marking) and is used in accordance with the instructions of screening procedure.

2.3 Competent Ethics Committee (CEC)

Commission Cantonale d’éthique sur la recherche sur l'être humain (Lausanne)

2.4 Ethical Conduct of the Study

The study will be carried out in accordance to the protocol and to principles enunciated in the current version of the Declaration of Helsinki, the guidelines of Good Clinical Practice (GCP) issued by ICH. It follows the instruction of the European Directive on medical devices 93/42/EEC and the ISO Norm 14155 and ISO 14971, the Swiss Law and Swiss regulatory authority’s requirements.

2.5 Declaration of interest

The trial is supported by an unrestricted grant from the *Fonds Scientifique Cardiovasculaire Fribourg*.

2.6 Patient Information and Informed Consent

Detailed patient information and a written informed consent form will be provided to each participant prior to study enrollement. Patients will be given sufficient time (up to 24 hours) to consider

participation and the opportunity to personally converse with one of the participating physicians in case of uncertainties with regard to any aspect of the trial. Once the written informed consent is provided by the patient, he will be enrolled and randomized to one of the treatment groups. Patients can withdraw their consent unconditionally, at any time and without any justification. Medical data and biological material (blood samples) that have been collected to date will, however, be analyzed.

2.7 Participant privacy and confidentiality

The investigators affirm and uphold the principle of the participant's right to privacy and that they shall comply with applicable privacy laws. Especially, anonymity of the participants will be guaranteed when presenting the data at scientific meetings or publishing them in scientific journals.
Individual subject medical information obtained as a result of this study is considered confidential and disclosure to third parties is prohibited.

2.8 Early termination of the study

The Sponsor-Investigator (and any competent authority) may terminate the study prematurely according to certain unforeseen circumstances. These may include but are not limited to:

- ethical concerns,
- insufficient participant recruitment,
- when the safety of the participants is doubtful or at risk, respectively,
- alterations in accepted clinical practice that make the continuation of a clinical trial unwise,
- early evidence of benefit or harm of the experimental intervention

2.9 Protocol amendments

The principal investigator as well as all co-investigators are entitled to amend the study protocol. The principal investigator is responsible for communicating important protocol modifications to the ethics committee as well as to the competent authorities including the clinical trial registry (clinicaltrials.gov).

3. BACKGROUND AND RATIONALE

3.1 Background and Rationale

Atrial Fibrillation (AF) is the most commonly encountered sustained arrhythmia in the outpatient or in-hospital setting (1) (2). It is estimated that 3% of the total population above the age of 20 suffers from some form of AF (Bjorck S; Stroke 2013). The prevalence of AF increases with increasing age. Furthermore and due to shifting demographics, better detection of silent AF and increasing prevalences of cardiovascular disorders predisposing its occurrence, the prevalence of AF is constantly waxing (3). The prevalence of AF in the hospital setting has been reported at variable rates: as low as 10%(4) and up to 31.3% (5). Camapnini and colleagues e.g. found AF in 18.2 % (6) of patients admitted to a general internal medicine ward.

The burden AF inficts on public health is considerable. Overall mortality rates in AF patients are almost twofold compared to the general population. The morbidity associted with AF is mainly related to its consequences such as stroke, worsening heart failure or white matter lesions in the brain leading to cognitive impairment. It is estimated that 10-40% of all AF patients are hospitalized at least once every year (7).

The clinical symptoms associated with AF are palpitations, chest pain and a reduction in exercise tolerance. Nevertheless, approximately 25% to 30% of AF patients are asymptomatic. Recent studies have shown that silent AF confers an increased risk for stroke similar to the risk incurred with permanent atrial fibrillation (8). Moreover, identification of unknown AF in the general population and subsequent anti-thrombotic treatment could reduce the overall stroke burden (9). Therefore it seems crucial to detect silent AF in the general population before the occurrence of stroke or systemic embolisms.

The current ESC-Guidelines (2016) recommend opportunistic screening for AF by pulse taking or ECG in all patients >65 years (10) (11). These same guidelines encourage the further evaluation of systematic AF screening programmes in at-risk populations. Several studies examined optimal AF screening methods in the ambulatory setting. The STROKESTOP study (9) screened 7173 individuals with an intermittent 1-lead ECG recording over 3 weeks detecting previously unknown AF in 3 % of the studied population. However, no systematic screening methods using new technologies have been formally evaluated in the hospital setting. Therefore, the objective of Detect-AF is to evaluate the efficacy of systematic AF screening with a one-lead ECG handheld device (Zenicor) in hospitalized patients.

3.2 Investigational Product and Indication

3.2.1 Investigational Product

The device is a one-lead ECG system and is intended to be used in hospitals and non-clinical environments. The intended use of the device is to take ECG measurements from the thumbs of the patient, to store and subsequently transmit the corresponding data to a central server for clinical analysis.

Zenicor-ECG by Zenicor Medical Systems AB is comprised of:
- Zenicor ECG-2 devices with software version 1.0060.
- Zenicor-ECG Back-end system, is an accessory to Zenicor ECG-2. The back-end system is branded as “Zenicor ECG Doctor system”. System version 3.2.

Both ECG-2 and Back-end system are CE marked. Product classification according to MDD 93/42/EEC: IIa

Intended use ECG-2:
The intended use of the device is to take electrocardiogram measurements from the thumbs of the patient, and to store and subsequently transmit the corresponding data to a central server for clinical analysis. The device constitutes a one-lead ambulatory ECG system, and is intended to be used in hospitals and non-clinical environments.

Intended use Zenicor-ECG Back-end system:
The intended use for the Back-end system is to display ECGs, to provide support for the interpretation and analysis of the measuring data from the Zenicor ECG-2 device. The system is intended to store ECG measurements and patient data as well as assign the devices with the patients. The users, which are medically trained personnel, can make a decision based on the displayed results. The Back-end system is intended to be used to set the diagnosis for patients of all ages with a suspected cardiac arrhythmia and to be used in screening investigations in order to prevent strokes.

3.2.2 Training

Care provider: 1 hour of initial training is provided before starting to use the device and is generally considered as sufficient for use of the device. Additional training will be provided upon request.
For the patient, there is no training required other than oral instruction at the time of use from the caregiver.

3.2.3 Contact with body

The Zenicor ECG device makes skin-contact to the thumbs of the patient via the electrodes. The electrodes are made of standard medical grade AgCl-ceramic.

3.3 Clinical Evidence to Date

The device has been on market since 2010. No recall has been made. Over 25 published scientific studies and 4 Doctoral theses have been made using the Zenicor device. A complete list of references can be foudn on the Zenicor website (https://zenicor.com/list-of-references-2/).

3.4 Medical Device: Rationale for the intended purpose in study

The product is in regular use in over 350 clinics in Europe and has been validated in more than 25 scientific publications and four Doctoral theses.

3.5 Explanation for choice of comparator (or placebo)

There will be no systematic AF screening in patients assigned to the control group. In these patients, pulse taking and ECG-recordings will be performed according to the treating physicians' clinical judgment. As a general rule, all patients hospitalized in Internal Medicine at Hospital Fribourg will have their vital signs taken at least once daily by trained nurses. Additional screening will be performed if the patient presents signs and symtoms suggestive of or consistent with AF.
This trial assesses the efficacy of a screening method. In our opinion, there is no need for a sham to control the screening method.

3.6 Risks / Benefits

Guidelines strongly and uniformly recommend anticoagulation in patients with AF in order to reduce the thromboembolic risk. The benefits of oral anticoagulant therapy are directly proportional to the underlying stroke risk as measured by hypertension, age, diabetes mellitus, congestive heart failure, prior stroke, vascular disease, and gender (CHA2DS2 -VASc score). Despite the documented benefits of anticoagulation in high-risk patients, bleeding complications are a common concern with the use of anticoagulant. The factors associated with a higher bleeding risk are similar to those conferring a higher thromboembolic risk. Nevertheless, it is the patients with the highest thromboembolic risk that benefit the most from oral anticoagulat therapy.

A potential risk of the Detect-AF study is the overdiagnosis of atrial fibrillation. This might lead to a non-indicated anticoagulant treatment and might expose the patients to an unnecessarily increased bleeding risk.

However, every case of new AF diagnosed by use of the screening method will be reviewed by two independent cardiologists. In case of uncertainty, additional recordings, e.g. Holter or R-Test will be performed.

3.7 Justification of choice of study population

The 2016 ESC-Guidelines for the management of atrial fibrillation underscore the importance of the

evaluation of systematic AF screening in at-risk populations. Hospitalized patients consitute such an at-risk population because of their older age and their often multiple comorbidities predisposing to AF. To our knowledge, this is the first study evaluating a systematic screening method to detect atrial fibrillation in hospitalized patients.

4. STUDY OBJECTIVES

4.1 Overall Objective

The aim of this study is to assess the efficacy of a one-lead ECG handheld device (Zenicor) for the detection of previously unknown atrial fibrillation (AF).

4.2 Primary Objective

The primary objective is to assess the detection rate of atrial fibrillation with a one-lead ECG handheld device (Zenicor) in hospitalized patients.

4.3 Secondary Objectives

Secondary objectives are to assess the impact of AF screening on clinical endpoints such as death, ischemic stroke, systemic embolism and myocardial infarction.

5. STUDY OUTCOMES

5.1 Primary Outcome

The primary outcome is the proportion of newly detected atrial fibrillation in percent.

5.2 Secondary Outcomes

Secondary outcomes are death, cardiovascular death, stroke, transient ischemic attack or systemic embolism, myocardial infarction and bleeding at 2, 5, and 10 years after study enrollment.

6. STUDY DESIGN

6.1 General study design and justification of design

This is a single center, open label, randomized control trial (RCT) with an allocation ratio of 1:1. All patients admitted to the Internal Medicine Department of Hospital Fribourg will be screened for study enrollment. After enrollment, patients will be randomized to either the screening or the control group.

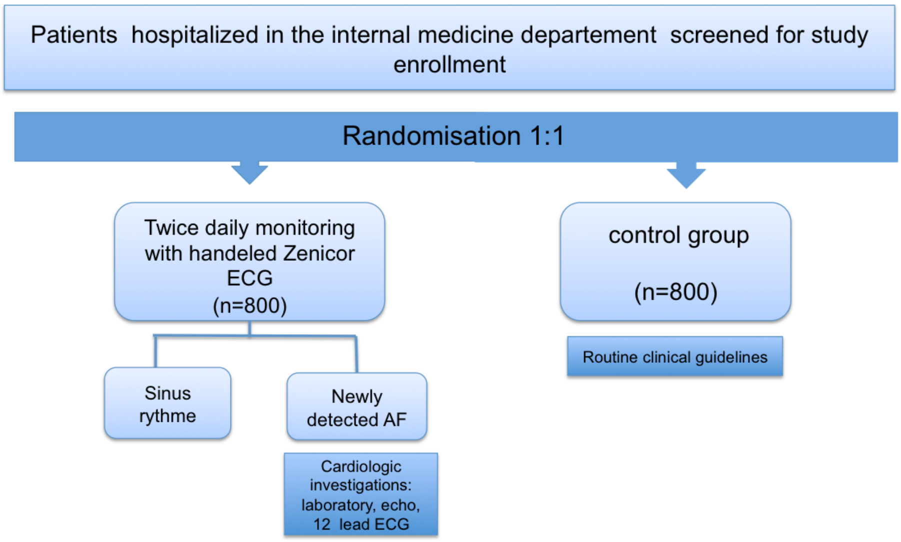

6.2 Methods of minimising bias

6.2.1 Randomisation

Participants will be randomized 1:1 to either the screening or the control group via a computer generated randomization sequence (www.randomizer.org).

6.2.2 Blinding procedures

There is no blinding in the present study. We jugde the employment of a sham-controlled screening unnecessary.

7. STUDY POPULATION

7.1 Eligibility criteria

All patients admitted to the Internal Medicine Department at Hospital Fribourg throughout the study period will be screened for study enrollment. Patients with known or previously documented AF, patients with a cardiac pacemaker, and implantable cardioverter-defibrillator or intra-cardiac monitoring device will be excluded from the present trial. Patients with an estimated life expectancy of < 6 months will likewise be excluded. Patients unable or unwilling to provide written-informed consent, to participate in the study procedures or in clinical follow-up will are excluded from the trial.

7.2 Recruitment and screening

Study nurses will screen all patients admitted to the Internal Medicine Department at Hospital Fribourg throughout the study period for eligibility. If the eligibility criteria are met the patient will be formally enrolled. The written-informed consent form will be signed by the patient as well as by one of the participating physicians who is at the patient’s disposition should the latter have any questions regarding to the trial.

7.3 Assignment to study groups

Randomization will be performed using a computer generated random list of numbers. A research nurse will assign the patients to the study groups according to the previously generated randomization sequence after study enrollment.

7.4 Criteria for withdrawal / discontinuation of participants

Patients can withdraw from the study at any given time and without providing any reason at all. Patients may be discontinuated from the trial if they refuse to comply with the study intervention.

8. STUDY INTERVENTION

8.1 Identity of Investigational Products (treatment / medical device)

8.1.1 Experimental Intervention (treatment / medical device)

Participants allocated to the treatment group will place their thumbs on the device twice daily and whenever they notice palpitations. Recordings will be obtained in the presence of specially trained nurses. The recordings will then be validated through a web-based interface operated by Zenicor (Zenicor-ECG Doctor System). Each one-lead ECG will be reviewed by the investigators via the Web-based analysis service (Zenicor-ECG Doctor System) to assess the presence of AF.

8.1.2 Control Intervention (standard/routine treatment / medical device)

Patients allocated to the control group will not undergo systematic monitoring of AF. In those patients, recording of 12 lead ECG or any other monitoring modalities will be employed according to routine clinical practice.

8.1.3 Packaging, Labelling and Supply (re-supply)

The Zenicor-ECG is delivered with free carrier on site at the time of the education.
Each device is labelled with a unique serial number, example A002130.

8.2 Medical Device Accountability

During the trial period Zenicor is accountable for any damage to the devices. Broken or damaged device will be sent to Zenicor for service or destruction. A replacement device will be sent to the customer during the service period or as replacement for the broken/lost device.

8.3 Return or Destruction of Medical Device

Devices can be returned by mail in a padded box to Zenicor after the study or purchased for further use in the clinic.

9. STUDY ASSESSMENTS

9.1 Study flow chart(s)/table of study procedures and assessments

	Patient information and inclusion[2]	*Intervention Period*	*Investigations*	*Follow-up*
Visit	*Once or twice*	*twice daily*	*In patients with newly diagnosed AF*	*2 years,<br>5 years,<br>10 years*
Time	*20 minutes*	*30 seconds twice daily (or more)*	*30 minutes*	
Patient information	*x*			
Informed Consent	*x*			
Baseline characteristics: age, gender, CHAD-VASC score etc.	*x*			
Randomisation	*x*			
ECG recordings		*x*		
Primary outcome		*x*		
Laboratory tests: TSH ,lipid profile etc.			*x*	
ECG, Echo			*x*	
Secondary outcome (clinical follow-up)				*x*

[2] A period of 24 hours of reflection will be granted to the patient for the constent process

9.2 Assessments of outcomes

9.2.1 Assessment of primary outcome

The primary end point will be the proportion of newly detected atrial fibrillation.

Recording will be done twice daily in the presence of specially trained nurses. In case of bad quality, the measurement will be repeated. The recordings will be validated through a web-based interface offered by Zenicor. The one-lead ECGs will be reviewed by the investigating physicians through the Web-based analysis service (Zenicor-ECG Doctor System) to assess the presence of AF.

The presence of an atrial fibrillation is defined as an irregularly irregular rythm in the abscence of discernable p waves or the presence of f-waves in at least one of the ECG registration.

9.2.2 Assessment of secondary outcomes

The secondary outcomes are the incidence of ischaemic stroke or systemic embolism and myocardial infarction. Information about secondary outcomes will be identified during a follow-up visit at 2,5 and 10 years.

Patients will be called by the study invastigator. A structured script will be used to guide a conversation to obtain information on secondary outcomes. Information about deaths occurring during the study period will be obtained from the Swiss population register.

9.3 Procedures at each visit

9.3.1 *Baseline visit*

A research nurse will evaluate every patient in the medicine department of the hospital Fribourg for the for study enrollment according to the exclusion criteria. Selected patients will receive written and verbal information from the study nurse and then be invited to sign a written consent.

Baseline assessment of all study patients will include the following: a analysis of patient demographics (age, gender), concomitant diseases (hypertension, dyslipidemia, diabetes, heart failure, vascular disease, previous stroke/TIA/thrombo-embolism), calculation of clinical score for atrial fibrillation stroke risk (CHA_2DS_2-VASc score), and previous symptoms probably related to undetected paroxysmal AF (such as intermittent breathlessness, palpitations, chest pain).

9.3.2 Daily visit

Patients allocated to the treatment group will undergo daily monitoring with the handheld Zenicor ECG. Participants will place their thumbs on the device at least twice daily and whenever they notice palpitations.

9.3.3 Cardiological visit

All individuals with newly detected AF are offered a individualized medical visit by a cardiologist to assure an adequate treatment. Individual net benefit from OAC treatment will be assessed by a 12

lead-ECG, echocardiography and blood sample analysis (with renal function, electrolytes, lipid profile).

9.3.4 Follow-up

Follow-up will be carried out at 2, 5 and 10 years after the diagnostic by a medical interview by telephone. The following data will be assessed: ischemic stroke or systemic embolism and myocardial infarction.

10. SAFETY

10.1 Definition and Assessment of safety related events

Active screening for AF can lead to both false positive results and false negative results.
It can increase anxiety and costs for further diagnostics in false positive cases or provide a false sense of security and lead to complacency relating to AF-related symptoms in false negative cases.

10.2 Reporting of Safety related events

Health hazard that require measures will be reported to the Sponsor-Investigator within 24 hours upon becoming aware of the event and within 2 days to the local Ethics Committee.

11. STATISTICAL METHODS

Categorical variables will be reported as counts and percentages; continuous variables will be reported as mean and SD or as median with 25% to 75% interquartile range according to their distribution. Normality will be assessed by visual inspection of histograms, the computation of Q-Q plots and the shapiro-wilk test. Categorical variables will be compared using chi-square or Fisher's exact test as appropriate. Continuous variables will be analyzed using the Student's t-test or the Wilcoxon rank-sum test according to their distribution. Survival free from the occurrence of clinical end points will be compared using the log-rank test and plotted as Kaplan-Meier survival functions. All statistical analyses will be performed using dedicated software (Stata version 13, StataCorp LP, College Station, Texas) at a 2-tailed significance level of alpha = 0.05.

11.1 Hypothesis

The null hypothesis to be rejected is that there is no difference with regard to the percentage of newly detected AF in the control and the intervention group.

11.2 Determination of Sample Size

Based on previous research, the expected annual detection rate is 3% in the Zenicor group and 1% in the control group. The inclusion of n=745 patients per group would yield a power of 80% and a significance of alpha = 0.05 to detect that difference. In order to account for ineffective measurements, post-inclusion drop-outs or other unforeseen drop-outs, we seek to include n=800 patients per group.

11.3 Planned Analyses

11.3.1 Primary Analysis

The primary analysis pertains to the proportion of newly detected atrial fibrillation. The primary analysis will compare the proportion of newly detected AF between the intervention and the control group. Univariate methods (Chi^2-Test) will be employed. If a significant difference in baseline characteristics will be observed, then logistic regression will be perform in order to correct for baseline imbalanaces.

11.3.2 Secondary Analyses

A secondary analysis will focus on subgroups. Pre-specified subgroups comprise gender, age, diabetes, hypertension, ischemic heart diseease, congestive heart disease and all subcategories of the CHADS-Vasc Score. Additional analysis will be performed on clinical follow-up. Event-free survival beteween the intervention and the control group will be compared at 2, 5 and 10 years according to the Kaplan-Meier method. Adjustment for eventual imbalances will be performed by using Cox

proportional hazards given that the data do not violate the proportionality assumption.

11.3.3 Interim analyses

A planned interim analysis will be carried at mid point of the trial (after half the patient had been entered and completed). The interim analysis will solely concern itself with the primary end point and will compare the proportion of newly detected AF between the study groups.

11.3.4 Deviation(s) from the original statistical plan

All deviations from the original statistical analysis plan will be provided in the final clinical study report.

11.4 Handling of missing data and drop-outs

Missing data will be ommitted pairwise from the analyses. Whenever data is missing, it will be explicitly stated in the final results of the study. Missing data arising from a loss to follow-up will be censored. The number of drop-outs will be provided in a flow-chart along with the reasons for said drop-out.

12. QUALITY ASSURANCE AND CONTROL

The principal investigator will be responsible for proper training of all involved study personnel. All involved personal at University and Hospital Fribourg will by trained by the Zenicor Incorporation before starting to use the device. Additional training will be provided upon request.

12.1 Data handling and record keeping / archiving

During the clinical trial, data will be accurately recorded and the original documents will be archived at the clinical trials unit at University and Hospital Fribourg. At the end of the study, Zenicor ECG will be saved in PDF form and stored for 10 years in the Zenicor-EKG Back-end system. All data collected will not include identifiable references to the subjects. The subject’s anonymity will be maintained and the confidentiality of records and documents that could identify subjects will be protected.

12.1.1 Case Report Forms

Patient characteristics, procedural characteristics, study outcomes and clinical follow-up will be recorded on electronical Case Report Forms (REDCap). For each enrolled study participant one single CRF is drafted.

12.1.2 Specification of source documents

Source document will contain demographic data, cardiovascular risk factors, laboratory results and informations about the follow up. CRF, Informed Consent Forms, Zenicor ECG and echocardiography will be found at the site of study.

12.1.3 Record keeping / archiving

1 lead-ECG will be stored in the Zenicor system throughout the whole study. Individual reports for each patient will be downloaded as pdf, including all or selected ECG's and will be exported for local archiving.

12.2 Data management

12.2.1 Data Management System

Patients data will be transcribed with RED cap Software to create electronic CRF, designed to capture study information. A trained study nurse will complete data management procedures, including coding through a computer generating de-identified number (random.org), verification, validation, security and storage of the database.

12.2.2 Analysis

Analysis of data will be performed using STATA MP 13 (Stata Inc., College Station, TX, USA).

12.2.3 Electronic and central data validation

The Zenicor ECG will be transmitted to a central server for clinical analysis of the data. The investigators will interpret the validity and diagnosis on the displayed results.

12.3 Monitoring

The following assessment for a minimal risk study are completed:

- Study poses no more risk than expected in daily life (no risk in the skin-contact to the thumbs of the patient to the zenicor electrodes, routine blood draw, physical exam).
- No vulnerable population included
- Trial procedures is not clinically unusual and complex

A possible misinterpretation of Zenicor electrocardiograms could lead to diagnotic errors and to unadapted treatment. The zenicor method has shown a sensitivity of 96% and specificity of 92% in detection and diagnosis of atrial fibrillation when compared with 12-lead ECG. The somewhat lower specificity reflects that the device rather overdiagnose than neglect patients with atrial fibrillation.

However, each case of newly diagnosed atrial fibrillation by use of the Zenicor device will be examined by two independent cardiologists. In case of uncertainty, additional recordings (by Holter or R-Test) will be made. The risk of diagnotic errors is very close to the usual risk taken in routine pratice.

An external monitoring of the study will be carried out retrospectively and prospectively:

- 1st on-site visit, intermediate visits and closing visit

- study risk analysis
- verification of source data
- CRF verification
- revision of consent forms
- verification of the recruitment procedure
- validation of eligibility criteria
- evaluation of the main evaluation criterion
- evaluation of Serious Adverse Reaction Assessment (SAE)

12.4 Confidentiality, Data Protection

Data will be stored physically and electronically on the personal computers at the clinical trials unit at University and Hospital Fribourg. Physical data are protected by the restricted acces to their location with only the involved medical personal having acces to it. Electronical data are protected by the *IT-Services of Etat de Fribourg SITEL*. The investigators will have access the the protocol, dataset, and statistcal code, during and after the study for publication and dissemination. The study nurse will only have access to the dataset during the study time.

12.3.1 Data protection and Storage at Zenicor (SWEDEN)

All communication between the web browser and the Zenicor server is encrypted with 128 bits encryption with SSL (HTTPS). To login, user-ID and password is needed. All login attempts are logged with date and time. After 5 unsuccessful logins, the user-ID is blocked. The user can only view and access patients in the organization connected to the user. 2-factor authentication login can be provided upon request.

The communication from the server to internet is duplicated and protected by a firewall and a Web Application Firewall (WAF). The WAF filters, monitors and blocks HTTPS traffic to and from the web application. Administration of server is performed only by authorized personnel connecting via VPN.

All transactions in the database are logged in a transaction log. All changes in the ECG-database made by any user are logged by the server. If a user updates or deletes patient data and/or ECG recordings, the transactions are logged with date, time, user name and organizational unit. System logs are retained for 1 year.

The Zenicor server is located in an ISO27001 certified secure data center. The Data Center have physical access control, fire protection, climate control and equipment for uninterrupted power supply including use of independent electric generators.

The database of the master server is replicated to a slave server. A back-up of both master and slave is performed every 24h.

12.3.2 Security of data transmission between Fribourg and Sweden

All communication between the device and the back-end system is performed over a GPRS-link and sent as a HTTP POST. When the patient has recorded an ECG and request transmission of ECG, the

device will perform a start-up of the modem. The device then sets up a link to the GSM network. When link is up the device sends one or more measures. After each measurement is sent, the backend system (containing the ECG database) sends an acknowledgment that the ECG is successfully received to the device. When done sending all measurements, the modem is turned off.
If the device does not receive an acknowledgement from the backend system that transmission was successful, the device will re-send the ECG at the next requested transmission occasion. The device can store up to 200 unsent ECG's in case GPRS link cannot be established, data transmission fails to be acknowledged or if transmission is simply not requested by the patient.
The ECG is sent from the device as unprocessed raw data from the AD-converter. The raw measurement data has each measurement sample represented by 3 bytes, which is how the ADC encodes each measurement sample. All post-processing including signal processing and creation of an ECG image is performed by the backend system.
The ECG is sent without any patient identification information, but only device serial number. The device has no information of who is using the device, but only records, labels the ECG with the device serial number/time of recording and then transmits the ECG. The ECG is recognized by the backend system through the device serial number. As the backend system has the information of which patient is currently using the specific device ID, the ECG labeled with a corresponding device ID and time stamp can be connected to correct patient in the ECG database.

13. FUNDING AND SUPPORT

13.1 Budget

	Estimated cost in CHF
**Personnel Costs**	
Study Nurse 50%	60'000
**Devices and Equipements**	
Zenicor handheld ECG x12	12'000
Barcode reader x12	2'000
**Publication and Dissemination**	
Travel expenses for Dissemination	5'000
Congress fees	2'000
Estimated total cost	***81'000***

13.2 Funding

The trial will be funded by the *Fonds Scientifique Cardiovasculaire Fribourg*. All external public or private institutions contributing to the trial will provide unrestricted grants to the *Fonds Scientifique Cardiovasculaire Fribourg* during the study periode. However, these parties still need to be defined.

14. REFERENCES

1. Friberg L, Bergfeldt L. Atrial fibrillation prevalence revisited. J Intern Med. 2013 Nov;274(5):461–8.
2. Heeringa J, van der Kuip DAM, Hofman A, Kors JA, van Herpen G, Stricker BHC, et al. Prevalence, incidence and lifetime risk of atrial fibrillation: the Rotterdam study. Eur Heart J. 2006 Apr;27(8):949–53.
3. Krahn AD, Manfreda J, Tate RB, Mathewson FA, Cuddy TE. The natural history of atrial fibrillation: incidence, risk factors, and prognosis in the Manitoba Follow-Up Study. Am J Med. 1995 May;98(5):476–84.
4. Stewart FM, Singh Y, Persson S, Gamble GD, Braatvedt GD. Atrial fibrillation: prevalence and management in an acute general medical unit. Aust N Z J Med. 1999 Feb;29(1):51–8.
5. López Soto A, Formiga F, Bosch X, García Alegría J, en representación de los investigadores del estudio ESFINGE. [Prevalence of atrial fibrillation and related factors in hospitalized old patients: ESFINGE study]. Med Clin (Barc). 2012 Mar 17;138(6):231–7.
6. Campanini M, Frediani R, Artom A, Pinna G, Valerio A, La Regina M, et al. Real-world management of atrial fibrillation in Internal Medicine units: the FADOI “FALP” observational study. J Cardiovasc Med Hagerstown Md. 2013 Jan;14(1):26–34.
7. Kim MH, Johnston SS, Chu B-C, Dalal MR, Schulman KL. Estimation of total incremental health care costs in patients with atrial fibrillation in the United States. Circ Cardiovasc Qual Outcomes. 2011 May;4(3):313–20.
8. Healey JS, Connolly SJ, Gold MR, Israel CW, Van Gelder IC, Capucci A, et al. Subclinical atrial fibrillation and the risk of stroke. N Engl J Med. 2012 Jan 12;366(2):120–9.
9. Svennberg E, Engdahl J, Al-Khalili F, Friberg L, Frykman V, Rosenqvist M. Mass Screening for Untreated Atrial Fibrillation: The STROKESTOP Study. Circulation. 2015 Jun 23;131(25):2176–84.
10. Camm AJ, Lip GYH, De Caterina R, Savelieva I, Atar D, Hohnloser SH, et al. 2012 focused update of the ESC Guidelines for the management of atrial fibrillationAn update of the 2010 ESC Guidelines for the management of atrial fibrillationDeveloped with the special contribution of the European Heart Rhythm Association. Eur Heart J. 2012 Nov 1;33(21):2719–47.
11. Fitzmaurice DA, Hobbs FDR, Jowett S, Mant J, Murray ET, Holder R, et al. Screening versus routine practice in detection of atrial fibrillation in patients aged 65 or over: cluster randomised controlled trial. BMJ. 2007 Aug 25;335(7616):383.

15. APPENDICES

1. Technical overview Zenicor ECG

2. Investigator’s CV

3. Investigator’s proof of GCP training

4. Case Report Forms

5. Patient informed consent (D/F)
